# Results of Cochlear Implantation in Patients with Congenital Rubell—Retrospective Study

**DOI:** 10.3390/jcm14113999

**Published:** 2025-06-05

**Authors:** Aleksandra Kolodziejak, Natalia Czajka, Rita Zdanowicz, Henryk Skarżyński, Piotr Henryk Skarżyński

**Affiliations:** 1 Department of Teleaudiology and Screening, World Hearing Center, Institute of Physiology and Pathology of Hearing, 02-042 Warsaw, Poland; n.czajka@ifps.org.pl (N.C.); r.zdanowicz@ifps.org.pl (R.Z.); p.skarzynski@csim.pl (P.H.S.); 2 Otorhinolaryngosurgery Clinic, World Hearing Center, Institute of Physiology and Pathology of Hearing, 02-042 Warsaw, Poland; h.skarzynski@ifps.org.pl; 3 Institute of Sensory Organs, 05-830 Warsaw, Poland

**Keywords:** congenital rubella, cochlear implantation, hearing loss

## Abstract

**Background/Objectives:** Congenital rubella syndrome (CRS) is an infection caused by rubella virus transmitted to the fetus during pregnancy, which can cause congenital hearing loss. Cochlear implant can be an effective therapy in patients with severe to profound bilateral hearing loss. The aim of this study was to evaluate the benefits of cochlear implantation in patients with profound hearing loss caused by congenital rubella syndrome. **Methods**: In total, 38 patients with profound hearing loss caused by intrauterine rubella virus infection were considered for cochlear implantation. Patients ranged in age from 8 to 72 years on the day of surgery, with a mean age of 27 years and median of 25 years (*SD* = 13.2). Preoperatively, all patients underwent pure-tone audiometry, and free-field speech audiometry was conducted in a quiet environment with the patient wearing a fitted hearing aid. Postoperatively, patients underwent pure-tone audiometry to assess residual hearing, and free-field speech audiometry was conducted when the patients had an active implant. **Results**: The average preoperative hearing threshold (averaged across the seven frequencies from 0.125 to 8 kHz) was 99.2 dB HL (*SD* = 6.79), while the average postoperative hearing threshold was 103.4 dB HL (*SD* = 5.74). Twelve months after the operation, patients achieved a WRS in quiet scores ranging from 10% to 90%, with an average of 59.1% and median of 70% (*SD* = 25.8). **Conclusions**: Rubella during pregnancy can lead to severe congenital defects, with sensorineural hearing loss being the most common. Cochlear implants appear to be an effective treatment for profound hearing loss caused by congenital rubella syndrome.

## 1. Introduction

Rubella is an acute disease caused by the rubella virus, the sole member of the Rubivirus genus in the Togaviridae family [1,2]. Rubella is also known as German measles, from its first description by German clinicians. In 1914, Alfred Hess believed that rubella might be caused by a filtrable virus, as he did not observe bacteria in the blood of pediatric patients infected with the disease [3]. It was not until 1941 that Norman Gregg reported the association between congenital cataracts in newborns and maternal rubella infection during the first trimester of pregnancy [4,5]. Twenty years later, in 1962, the virus was isolated in culture by a research group, laying the foundation for vaccine development (Forrest et al., 1971) [6]. Clinically, rubella is a mild disease, initially causing flu-like symptoms (muscle pain, low-grade fever, loss of appetite), followed by a rash and painful lymphadenopathy [7]. However, if a woman is infected during the first trimester of pregnancy, it can cause a very adverse effect on the developing fetus and lead to congenital rubella syndrome (CRS) [8,9,10].

The World Health Organization (WHO) estimates that approximately 100,000 children are born each year with CRS [11]. Infection in a woman a few weeks before conception does not pose any risk to the fetus [12], but if the rubella virus infects a woman during the first 8–12 weeks of pregnancy, there is more than an 80% chance the baby will have congenital defects, such as mild to profound hearing loss, cataracts, heart defects, and intellectual disability [13]. There is also about a 20% risk of spontaneous abortion within the first 8 weeks of pregnancy [14,15].

The most common consequence of CRS is sensorineural hearing loss due to defects in the inner ear or auditory nerve [16,17]. Limited histopathological studies have revealed damage to cells in the cochlear duct and the stria vascularis of fetuses infected with the rubella virus [18]. These findings point to a specific explanation for hearing loss in infants with CRS [19], through the examination of the temporal bones of six infants with CRS. Researchers observed several pathological changes in the organ of Corti and atrophy of the saccule. A study by Ward et al. [20] noted the destruction of the stria vascularis.

Hearing loss can lead to impairments in speech, language development, and cognitive ability, making early detection crucial [21,22]. Depending on the degree of hearing loss, patients can be fitted with hearing aids or cochlear implants. In cases of severe to profound hearing loss, where conventional methods are ineffective, a cochlear implant should be considered [23,24,25].

The aim of this study was to evaluate the benefits of cochlear implantation in patients with profound hearing loss caused by congenital rubella syndrome.

## 2. Materials and Methods

### 2.1. Inclusion Criteria

Patients with profound hearing loss caused by intrauterine rubella virus infection were considered for cochlear implantation. The following inclusion criteria were applied: (1) average hearing threshold for frequencies of 0.5, 1, 2, and 4 kHz of 90 dB HL or worse, and bone conduction thresholds at the limit of the audiometer’s capabilities; (2) word recognition score with hearing aids below 50%; and (3) hearing loss due to congenital rubella virus. This study received approval from the Bioethics Committee (IFPS/Statement No. 2/2023) and was conducted in accordance with the Helsinki Declaration.

### 2.2. Patients

The study group consisted of 38 patients (22 female and 16 male) with profound hearing loss due to congenital rubella infection. Patients ranged in age from 8 to 72 years on the day of surgery, with a mean age of 27 years and median of 25 years (*SD* = 13.2). Such a late implantation had several causes: late diagnosis, lack of guidelines for managing patients after contracting rubella (including women who contracted it during pregnancy), and the fact that mandatory vaccinations were introduced in 1988. A late diagnosis could have impacted the outcomes achieved by the patients. In this group of patients, we did not observe patients with single-sided deafness (SSD).

### 2.3. Audiometric Evaluation

Preoperatively, all patients underwent pure-tone audiometry to determine air conduction and bone conduction thresholds across frequencies ranging from 125 to 8000 Hz. The same assessment was also conducted 12 months postoperatively in order to evaluate the preservation of residual hearing. Free-field pure-tone audiometry was also performed 12 months postoperatively. Standard speech audiometry was also performed preoperatively using the Demenko and Pruszewicz lists [26]. Another preoperative test conducted was free-field speech audiometry, in a quiet environment with the patient wearing a fitted hearing aid and sitting in front of a loudspeaker more than 1 m in front of them. Speech material from the Demenko and Pruszewicz lists was presented at 70 dB HL. The result of the test was the percentage of correctly repeated words (the word recognition score, WRS).

Twelve months after the surgery, a similar test was conducted when the patient had an active implant during the test. The speech material was presented at 65 dB HL both in quiet and in noise with 10 dB SNR.

### 2.4. Surgical Procedure

Surgery was performed under general anesthesia. The first step involved antromastoidectomy to open up the mastoid cavity, during which a piece of cortical layer was removed with a chisel and hammer. This fragment would later be used to isolate the mastoid cavity from the subcutaneous space, where the internal part of the implant needs to be placed. We found that in cases of rubella, the mastoid appeared normal (the virus did not cause any changes in this area). Cutting with a diamond burr required no special procedure, and enlarging the cavity was not required. The second stage involved posterior tympanotomy (with preservation of the bony bridge over the body of the incus and posterior to the drilled area) until the round window niche was visualized. A 1.8 mm diamond burr was standard, but sometimes a 1.4 or 2.3 mm burr was used. Where there was limited access to the round window, a 0.8–1.0 mm diamond burr was used to drill out part of the bony overhang. The third stage involved making an incision in the round window membrane through which the electrode array was inserted into scala tympani. Then, the area around the electrode at the round window area was sealed and secured in place using tissue or a small piece of periosteum, sponge with antibiotic, and tissue glue. Finally, the receiver was secured to the bone in the periosteal pocket, and the skin behind the ear was triple sutured [27,28,29].

## 3. Results

### 3.1. Clinical Characteristic of the Study Group

The average duration of hearing loss was 27.1 years. Most patients had used hearing aids prior to implantation. They first received hearing aids when they were aged 0.42 to 8 years (average 2.62 years, *SD* = 1.87). Most patients (58%) had undergone a CT scan before surgery. Most patients had received a Med-El cochlear implant (71%), most commonly a Flex28 electrode (26%). Details are shown in Table 1.

### 3.2. Comparison of Preoperative and Postoperative Hearing Thresholds

**Figure 1:**
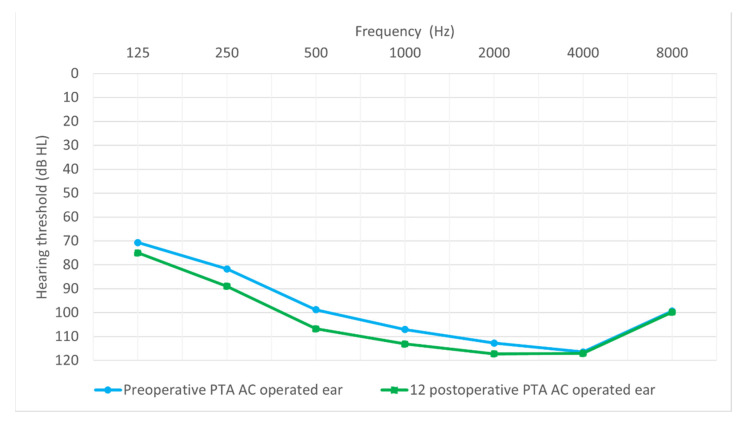
Preoperative and 12-month postoperative average hearing thresholds for the operated ear.

**Figure 2:**
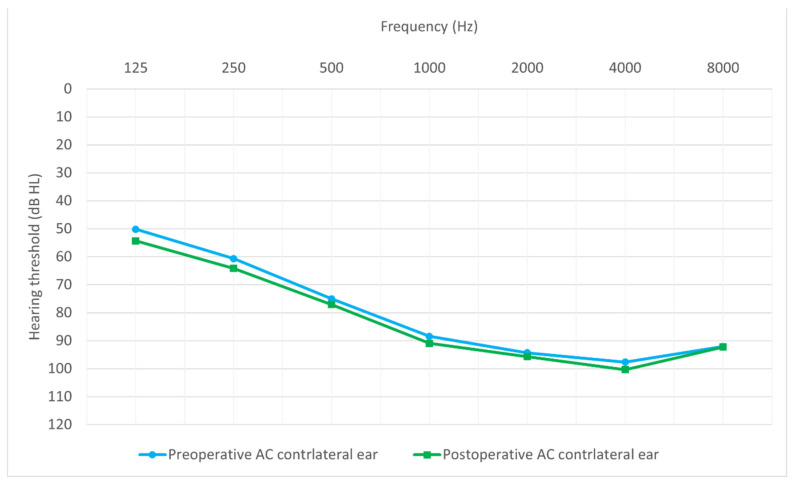
Preoperative and 12-month postoperative average hearing thresholds for the contralateral ear.

Figure 1 shows the average hearing thresholds of the operated ear for all patients obtained by tonal audiometry from 0.125 to 8 kHz, both preoperatively and 12 months postoperatively. The average hearing thresholds for the non-operated ear, measured at the same time points, are presented in Figure 2.

Specifically, the average hearing threshold, for 125 Hz, before surgery was *M* = 78.68 dB HL (*SD* = 12.23), while after surgery, it was *M* = 82.5 dB HL (*SD* = 9.85). The average change was 3.82 dB HL, and it was not statistically significant: *Z* = 1.84; *p* = 0.065. The average hearing threshold, for 250 Hz, before surgery was *M* = 86.71 dB HL (*SD* = 11.47), while after surgery, it was *M* = 92.11 dB HL (*SD* = 8.75). The average change was 5.4 dB Hz, and it was statistically significant: *Z* = 2.73; *p* = 0.006. The average hearing threshold, for 500 Hz, before surgery was *M* = 97.11 dB HL (*SD* = 10.11), while after surgery, it was *M* = 103.42 dB HL (*SD* = 12.47). The average change was 6.32 dB HL, and it was statistically significant: *Z* = 2.44; *p* = 0.015. The average hearing threshold, for 1000 Hz, before surgery was *M* = 107.11 dB HL (*SD* = 8.67), while after surgery, it was *M* = 112.89 dB HL (*SD* = 8.02). The average change was 5.79 dB HL, and it was statistically significant: *Z* = 3.2; *p* = 0.001. The average hearing threshold, for 2000 Hz, before surgery was *M* = 111 dB HL (*SD* = 11.2), while after surgery, it was *M* = 116 dB HL (*SD* = 8.4). The average change was 4.9 dB HL and statistically significant (*Z* = 2.41; *p* = 0.016). Before surgery, the average hearing threshold for 4000 Hz was *M* = 111 dB HL (*SD* = 12.6), while after surgery, it was *M* = 117 dB HL (*SD* = 7.8). The average change was 5.66 dB HL and statistically significant (*Z* = 3.23; *p* = 0.001). For 8000 Hz, the average hearing threshold before surgery was *M* = 99 dB HL (*SD* = 8.8), while after surgery, it was *M* = 100 dB HL (*SD* = 1.6). The average change was 1.1 dB HL, and it was statistically significant (*Z* = 2.06; *p* = 0.039).

The average preoperative hearing threshold (averaged across the seven frequencies from 0.125 to 8 kHz was 99.2 dB HL (*SD* = 6.79), while the average postoperative hearing threshold was 103.4 dB HL (*SD* = 5.74). The average change was 4.7 dB and statistically significant (*Z* = 3.68; *p* < 0.001).

**Figure 3:**
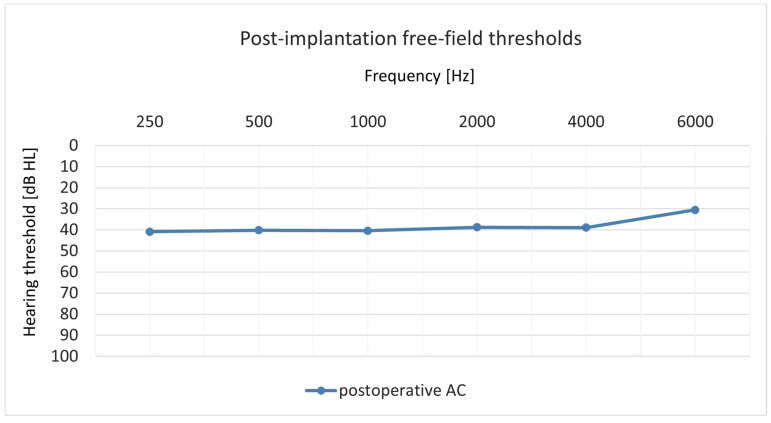
Averaged free-field pure-tone audiometry test results.

Twelve months after surgery, all patients underwent free-field pure-tone audiometry test. Figure 3 presents the averaged results of the patients.

### 3.3. Speech Understanding

Preoperatively, all patients (*n* = 38) completed a word recognition score (WRS) test under free-field conditions with their hearing aids and a sound level of 70 dB SPL. WRSs ranged from zero to 40% (*M =* 3.3%; *SD* = 7.38). Most patients (25) scored zero, with the others receiving scores of 5 to 40% (see Figure 3).

**Figure 4:**
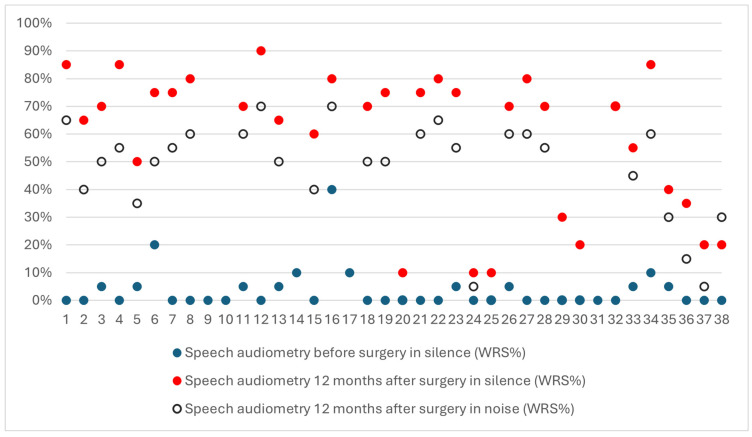
Preoperative and postoperative results of speech audiometry.

Twelve months after the operation, a similar WRS test was performed, this time with the cochlear implants active. During the test, the non-operated ear was plugged with a Peltor (3^TM^ PELTOR^TM^, 3M India Ltd., Bangalore, India). The sound level was set at 65 dB HL. In quiet conditions, patients achieved WRSs ranging from 10% to 90%, with an average of 59.1% and median of 70% (*SD* = 25.8). These results were higher than the preoperative scores, with the difference being statistically significant (*Z* = 4.86; *p* < 0.001). In noisy conditions (SNR = 10 dB), patients achieved WRSs from 0% to 70%, with an average of 42.5% and median of 50% (*SD* = 24.3). The individual results are shown in Figure 4.

### 3.4. Hearing Preservation

Hearing preservation scores were calculated for all patients. The minimum preservation of residual hearing was 5.7, and the maximum was 98.9, with a mean of 53.1 (*SD* = 31.4). Three patients who had total deafness preoperatively (i.e., no residual hearing) were not included in this analysis. Data on hearing preservation, categorized into minimal, partial, and total, are presented in Table 2. Most of the patients had partially preserved hearing (43%).

## 4. Discussion

The objective of this study was to evaluate the benefits of cochlear implantation in patients with profound hearing loss caused by congenital rubella virus infection. These patients had not received any significant improvement with conventional hearing aids, making cochlear implantation the only viable solution for restoring their hearing.

Our results show that the average preoperative hearing threshold was 99.2 dB HL for air conduction. In the free-field word recognition test, conducted under quiet conditions with hearing aids, patients were able to recognize an average of 3% of words, with 23 out of 38 patients not recognizing any words. After cochlear implantation, there was an improvement in speech understanding. Patients recognized an average of 59% of one-syllable words in quiet and 43% in noise.

Octaviani et al. [30] conducted a study on the benefits of cochlear implants in 11 patients with CRS. They used the CAP-II (Categories of Auditory Performance-II) scale [31] to assess the benefits. Preoperatively, five patients were in Group 1, indicating they had no awareness of environmental sounds. Twelve months after surgery, most patients were in Groups 6 and 7, which indicated, for Group 6, understanding conversation without lipreading with a familiar talker; and for Group 7, being able to use the telephone with a familiar talker. Toizumi et al. [32] studied 41 children with CRS, focusing on developmental delays and sensory defects. Hearing impairment was confirmed in 21 patients through an ABR test. One child underwent bilateral cochlear implantation and, following intensive rehabilitation, began to show auditory responses and develop speech. Pramila et al. [33] reported on a child in India with congenital rubella who had severe hearing loss accompanied by heart defects, retinopathy, and cognitive disorders. The child had a cochlear implant at age 5, after which they could detect sound, understand speech, and communicate well. Calháu et al. [34] investigated the main etiological factors causing deafness among 200 cochlear implant users and found that rubella was the second most common cause of deafness (12%).

In summary, the results obtained from our patients are satisfactory. One year after surgery, they demonstrated significantly better speech understanding. Patients who, since birth, previously faced substantial difficulties functioning in society can now participate in the world of sounds. This means better psychological well-being, improved quality of life, and easier daily functioning [35,36,37].

## 5. Conclusions

Rubella during pregnancy can lead to severe congenital defects, with sensorineural hearing loss being the most common. Cochlear implants appear to be an effective treatment for profound hearing loss caused by congenital rubella syndrome. However, further research is necessary to compare pre- and post-implantation outcomes in these patients.

### Limitations

The results are based on a retrospective analysis of 38 patients. A limitation was the sole reliance on the results available in our system, with no possibility of addressing gaps in the data for some patients.

## Figures and Tables

**Table 1:** Clinical data of patients (*n* = 38).

Operated ear	Right	26 (68%)
	Left	12 (32%)
Tinnitus in operated ear	Yes	18 (47%)
	No	20 (53%)
Tinnitus in the opposite ear	Yes	9 (76%)
	No	29 (24%)
Vertigo	Yes	5 (13%)
	No	33 (87%)
Hearing aids (HAs)	Yes	35 (92%)
	No	3 (8%)
HAs from what age?	Range (years)	0.42–8
	*M* (*SD*)	2.62 (1.87)
Computed tomography	Yes	22 (58%)
	No	16 (42%)
Surgical approach	Posterior tympanotomy, round window	31 (82%)
	Posterior tympanotomy, cochleostomy	7 (18%)
Implant	Med-El	27 (71%)
	Advanced Bionics	2 (5%)
	Oticon	1 (3%)
	Cochlear	8 (21%)
Type of implant	Concerto	6 (16%)
	Sonata	14 (37%)
	Synchrony	4 (11%)
	Pulsar	3 (8%)
	HiRes 90 k	2 (5%)
	Neuro Zti	1 (3%)
	CI422	5 (13%)
	CI522	1 (3%)
	CI512	2 (5%)
Electrode	Standard	7 (18%)
	Medium	1 (3%)
	FlexSoft	4 (11%)
	Flex28	10 (26%)
	Flex26	5 (13%)
	SlimJ	2 (5%)
	Evo	1 (3%)
	CI 512	2 (5%)
	CI 422/CI 522	6 (16%)

**Table 2:** Hearing preservation 12 months after cochlear implant activation. Data given as the number of patients (percentage in parentheses).

	Minimal	Partial	Complete
Hearing preservation	8 (23%)	15 (43%)	12 (34%)

## Data Availability

The data that support the findings of this study are available from the corresponding author [A.K.], upon reasonable request.
